# The Effect of Experimental Protocol on the Toxicity of Saxitoxin in Mice

**DOI:** 10.3390/toxins15040290

**Published:** 2023-04-17

**Authors:** Sarah C. Finch, Michael J. Boundy, Nicola G. Webb, D. Tim Harwood

**Affiliations:** 1 AgResearch Ltd. Ruakura Research Centre, Private Bag 3123, Hamilton 3240, New Zealand; nikki.webb@agresearch.co.nz; 2 Cawthron Institute, Private Bag 2, Nelson 7042, New Zealand; michael.boundy@cawthron.org.nz (M.J.B.); tim.harwood@cawthron.org.nz (D.T.H.)

**Keywords:** PSP, toxicity testing, risk assessment, regulatory limits, shellfish toxin, PST

## Abstract

Regulatory limits for toxins in shellfish are required to ensure the health of consumers. However, these limits also impact the profitability of shellfish industries making it critical that they are fit for purpose. Since human toxicity data is rarely available, the setting of regulatory limits is dependent on animal data which can then be extrapolated for use in the assessment of human risk. The dependence on animal data to keep humans safe means that it is critical that the toxicity data used is robust and of high quality. Worldwide, the protocols used in toxicity testing are varied, making it hard to compare results and adding confusion over which results better reflect the true toxicity. In this study, we look at the effect of mouse gender, i.p. dose volume, mouse body weight and feeding protocols (both acute and sub-acute) on the toxicity of saxitoxin. This allowed the effect of different variables used in toxicity testing to be understood and showed that the feeding protocol used in both acute and sub-acute studies greatly influenced the toxicity of saxitoxin in mice. Therefore, the adoption of a standard protocol for the testing of shellfish toxins is recommended.

## 1. Introduction

Paralytic shellfish poisoning (PSP) is caused by the ingestion of filter-feeding shellfish contaminated with toxins produced by dinoflagellates of the genera *Alexandruim*, *Gymnodinium* and *Pyrodinium* [1,2,3]. This poisoning is characterized by numbness of the lips, muscle weakness and incoordination [4] and has been reported worldwide throughout history [5]. To protect the health of consumers, regulatory limits for paralytic shellfish toxins (PSTs) have been set in many countries. The European Union (EU) has a regulatory limit of 800 µg STX.2HCl eq/kg of shellfish flesh (Regulation (EC) No 853/2004 of the European Parliament and of the Council of 29 April 2004) [6] and the same limit is specified in the Codex Standard for Live and Raw Bivalve Molluscs (CODEXSTAN 292-2008, rev 2015) [7]. These standards specify the limit in terms of STX.2HCl equivalents because the major driver of PSP, saxitoxin, has over 50 structural analogues all of which have varying degrees of toxicity [8]. To generate a regulatory limit, which encompasses all of these compounds, the toxicity of each analogue can be determined and compared to that of saxitoxin. This comparison generates a toxicity equivalence factor (TEF) [9] which can be applied to the analytically determined concentration of each analogue to generate a µg STX.2HCl eq value. By totaling the concentrations of all analogues (expressed as µg STX.2HCl eq), a figure for the total PST concentration in the sample can be determined and used to assess risk.

To estimate the risk to consumers and guide decisions on maintaining and revising regulatory limits for shellfish toxins, bodies such as the European Food Safety Authority (EFSA), collect relevant toxicity information for consideration by expert scientific panels who then make recommendations [10]. Toxicity data is considered in the following order of importance: data from human cases, oral median lethal dose (LD_50_) in animals, intraperitoneal (i.p.) LD_50_ in animals, mouse bioassay and in vitro data [11]. To determine the dose rates associated with human cases of poisoning, three pieces of data are required: the concentration of toxins in remnant food, information on how much of the contaminated food was eaten and the body weight of the consumer. While some data associated with a human intoxication event may be collected, it is rare that all three necessary pieces of information are available. It is critical that remnant food be analyzed rather than shellfish retrospectively collected from the same harvesting area because PST concentrations in shellfish can quickly change and can be highly variable. Furthermore, the cooking process of the contaminated shellfish as well as what is consumed (broth and/or shellfish) has an impact on toxicity. For these reasons, human cases are seldom used in the setting of regulatory limits. The alternative is to extrapolate animal data to assess human risk which necessitates the use of safety factors. Generally, a ten-fold safety factor is used to account for species difference and a further ten-fold safety factor is used to account for possible variations in susceptibility within a human population [12].

Although human health is of paramount importance and is the highest priority in the setting of regulatory limits for toxins in shellfish, it should not be forgotten that the limits set have a huge impact on shellfish industries. For example, in New Zealand, the aquaculture industry is worth NZD 650 M (USD 0.5 B) and in 2019, 115,000 tonnes of bivalve shellfish were produced [13], making the shellfish industry of significant economic importance to this country. Getting the regulatory limits correct is therefore critical for both human health and commercial endeavors. High-quality and robust toxicity data are, therefore, of paramount importance.

In 2016, a FAO/WHO technical paper stated that there have been no reported human poisoning cases from the consumption of commercial shellfish in 50 years [11]. The regulation of PSTs in commercial shellfish samples using the current limit of 800 µg STX.2HCl eq/kg of shellfish flesh, therefore, appeared fit for the purpose. However, more recently (2019) it was reported that a woman developed symptoms consistent with PSP after the consumption of mussels [14]. Although low concentrations of STX were detected in a raw sample of the same batch of mussels (size of batch not reported) remnant food was not available for analysis, leading the authors to declare that PSTs could not be definitively implicated in this case [14]. On balance it therefore appears that the current regulatory limit is adequately protecting human health. A recent feeding study also supports the current PST regulatory limit. This study used a feeding protocol relevant to humans and fed groups of mice twice daily with a diet containing STX.2HCl for 21 days [15]. Dose rates of up to 730 µg/kg/day (1962 nmol/kg/day) were administered with no adverse effects observed. Using the 100-fold safety factor required to extrapolate animal data for use in human risk assessment results in a dose rate of 7.3 µg/kg/day. For a 70 kg human to reach this dose rate, they would have to consume 630 g of shellfish flesh contaminated at the current regulatory limit (800 µg STX.2HCl eq/kg). Since this quantity of shellfish exceeds the EFSA definition of a large portion size (400 g) [10], this provides further confidence in the current PST regulatory limit. The experiment above showed that the no observable adverse effect level (NOAEL) for STX.2HCl by oral administration is greater than 730 µg/kg/day (1962 nmol/kg/day). To determine the true NOAEL, higher dose rates of STX.2HCl would need to be administered to mice. In this study, using the mealtime feeding protocol, mice were fed diets containing higher concentrations of STX.2HCl.

Current protocols used to determine the toxicity of PSTs vary with little inter-lab consistency. For acute toxicity studies the administration method, gender, body weight (maturity) and state of alimentation (using fasted or non-fasted animals) are all possible points of difference. The current inconsistencies in the methods used for toxicity determinations are of concern as it is difficult to compare results and generate robust regulatory limits. In this study, the impact of different experimental protocols on the toxicity of STX were determined. On the basis of the results generated, a standard protocol for the testing of PSTs is proposed.

## 2. Results

### 2.1. Saxitoxin Feeding Study

**Figure 1:**
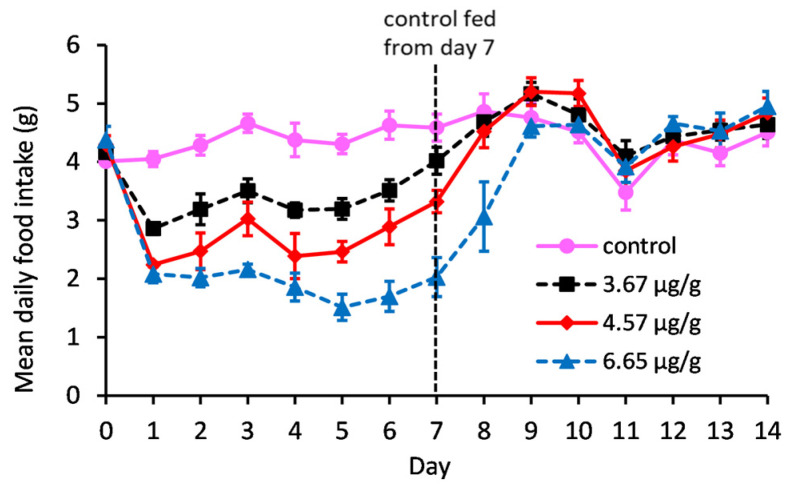
Mean daily food intake (g) of mice fed control, low STX (3.67 µg/g), med STX (4.57 µg/g) or high STX (6.65 µg/g) diets for 7 days followed by a further 7 days on control diet. Error bars represent SEMs.

The previous mouse feeding study saw no adverse effects of STX.2HCl at a daily dose rate of up to 730 µg/kg (1961 nmol/kg) using the mealtime feeding protocol. In the current study, higher concentrations of STX.2HCl were incorporated into the mouse diet with the hope that toxicity would be induced and thus allowing a NOAEL to be determined. This was unsuccessful as the food intake of mice decreased with increasing concentrations of STX.2HCl in the diet (Figure 1) and in fact, because of this effect, mice fed low, med and high STX-containing diets ingested similar dose rates of STX.2HCl (636 ± 14, 690 ± 31 and 755 ± 26 µg/kg/day) (1708 ± 37.6, 1853 ± 83.3 and 2028 ± 69.8 nmol/kg/day), respectively. Two mice in the high STX treatment group (one on day five and one on day six) showed symptoms of toxicity, including restricted movement, splaying of the back legs and a very low food intake. Since it was unlikely that these mice would survive any longer on their treatment diet, a control diet was fed to all mice starting on day seven. An immediate increase in food intake was observed for all mice in the STX treatment groups, although this effect was most marked for those in the high STX group (Figure 1). Within 24 h, the two mice who had exhibited toxicity were looking normal and eating well and within just 48 h, all mice had a similar food intake. 

### 2.2. The Effect of Dosing Protocols on the Toxicity of Saxitoxin

#### 2.2.1. The Effect of Mouse Bodyweight on the Toxicity of STX.2HCl by i.p.

The i.p. toxicity of STX.2HCl was not influenced by mouse bodyweight over a 14–25 g weight range (Table 1).

#### 2.2.2. The Effect of Dose Volume on the Toxicity of STX.2HCl by i.p.

The i.p. toxicity of STX.2HCl was consistent using dose volumes of 0.2–1 mL (Table 2). 

#### 2.2.3. The Effect of Mouse Gender on the Toxicity of STX.2HCl by i.p. and Orally

The median lethal dose rate of STX.2HCl was determined in male and female mice dosed i.p. and orally. This showed that gender had no influence on the toxicity of STX.2HCl by either route of administration (Table 3). Although both male and female mice are used in feeding studies, one gender is usually used in LD_50_ determinations, meaning that out of a litter of mice, one gender is often unused. The usage of both genders is therefore more ethically acceptable as the breeding required to generate enough mice is reduced.

#### 2.2.4. The Effect of Feeding Protocol on the Oral Toxicity of STX.2HCl

To determine the influence of the feeding regime on the oral toxicity of STX.2HCl LD_50_s were determined using several different feeding conditions (Table 4). As expected, the fasted mice were the most susceptible to the toxin. However, the large difference between the fasted mice and those with 0.5 g of mouse food in their stomach was somewhat surprising. This illustrates that this variable is critical to the accuracy and repeatability of oral toxicity results.

#### 2.2.5. The Effect of Dosing Method on the Oral Toxicity of Saxitoxin

The LD_50_ obtained by the gavage administration was 1.5 times lower than that determined when mice were dosed with STX.2HCl either on mouse food or in cream cheese. The LD_50_s determined by adding STX.2HCl to normal mouse food and by lacing cream cheese with STX.2HCl were the same (Table 5).

## 3. Discussion

Using the mealtime protocol, the previous study of feeding STX.2HCl to mice for 21 days showed that the NOAEL was greater than 730 µg/kg/day (1962 nmol/kg/day) as no adverse effects were observed at this dose rate [15]. To increase the dose rates of STX.2HCl, mice were fed diets containing three higher concentrations. After just one day of feeding, the food intake of mice showed a dose-dependent decrease and when normal food was introduced the low food intakes of the STX.2HCl treatment groups immediately increased. This immediate increase in food intake illustrates that the mice found the diet unpalatable, as opposed to not eating due to feeling ill. In the latter case, the reintroduction of normal mouse food would induce a more gradual increase in food intake. The first symptoms of PSP in humans include tingling on the tongue and numbness of the lips and mouth which can appear within minutes of chewing tainted shellfish. This effect is caused by the local absorption of toxins through the buccal mucous membranes in the mouth [16]. The palatability issue seen in this study is therefore likely to be due to the local absorption of STX.2HCl in the mouth, inducing an unpleasant response and deterring feeding. Although two mice fed the highest dose of STX.2HCl had symptoms of toxicity, this is not valid data for the calculation of a NOAEL. This is because these two mice were not eating, whereas other mice, with a higher food intake, were eating the same or greater dose rates of STX.2HCl with no adverse effects. This feeding experiment did not deliver the desired increase in STX.2HCl dose rate and a NOAEL could not be determined. However, it did demonstrate that mouse feeding is deterred by STX.2HCl and that the toxicity is significantly influenced by food intake. 

In acute toxicity studies i.p. injection, gender, mouse bodyweight and dose volume are possible variables in dosing protocol. Traditionally it has been believed that male mice are more susceptible to toxins compared to female mice due to absorption being less efficient in females [17]. However, the literature gives examples of both females and males being more susceptible to toxins with the magnitude of difference showing a large variation [17,18]. In this study we showed that there was no gender effect on the toxicity of STX.2HCl either by i.p. or oral administration. Although both male and female mice are currently used in feeding studies, only one gender is usually used in acute LD_50_ determinations. The use of both genders is more ethical, since less breeding would be required to provide the necessary mouse numbers for experimental work. 

It is generally accepted that human infants are more susceptible to toxins than adults and this has also been demonstrated in mice where, in comparison to adults, weanlings are more susceptible to some toxins [19]. Although many studies have shown a toxicity difference between weanling and adult mice [20], there is less information regarding the effect of more subtle differences in mouse weight. However, in one study the same i.p. dose rate of okadaic acid which was lethal to 100% of mice weighing 14–15 and 16–17 g was lethal to only 80% of mice weighing 19–20 g, 50% of mice weighing 21–22 g and 40% of mice weighing 23–24 g [21]. Currently, studies assessing the toxicity of shellfish compounds use a variety of body weight ranges. For example, different studies investigating the toxicity of yessotoxin used mice weighing 15–19 g [22], 16–20 g [23], 18–20 g [24] and 19.5–20.5 g [25] which, based on the okadaic acid result, has the potential to affect the toxicity result. However, our study has shown, that when administered by i.p. there is no effect of mouse bodyweight on the toxicity of STX.2HCl within a weight range of 14–25 g. Despite showing that there is no effect of mouse body weight on the toxicity of STX.2HCl, it is not safe to assume that human infants are not vulnerable to PSTs because age and maturity may also play a role in the intoxication.

In the original AOAC official mouse bioassay method for the detection of PSP toxins, a dose volume of 1 mL shellfish extract was administered i.p. to mice [26]. This may be the reason that multiple studies have still used a 1 mL injection volume [27,28,29], despite the fact that it exceeds the good practice guidelines of <0.5 mL [10]. In this study, it was demonstrated that the toxicity of STX.2HCl was consistent using injection volumes of 0.2–1 mL. It is therefore recommended that an injection volume of 0.2–0.5 mL be used for i.p injection, a volume which is in accord with good practice. However, the lack of an effect of dose volume on toxicity means that comparison with studies using larger injection volumes is still valid. 

The result of the STX.2HCl feeding study reported in this paper suggested that the amount of food in the stomach of mice at the time of dosing had a big impact on toxicity. There is considerable debate over whether mice should be fasted or not and if so, how long that fasting period should be. It could be argued that fasting will create the worst possible scenario where contaminated shellfish are eaten on an empty stomach [30]. However, when STX is consumed, it comes along with the shellfish matrix making it impossible to eat PSTs on an empty stomach. Using this artificial situation of a fasted mouse with an empty stomach, the toxicity of shellfish toxins is overestimated. Consistent with this observation, in this study, the toxicity of STX.2HCl was observed to be 1.9 times greater in a fasted mouse compared to one with unlimited access to food. Another argument for using fasted mice is to limit the variability which exists with ad lib feeding. This variability arises because bouts of feeding and the amount of food consumed at each feeding event will be different for each mouse yielding different quantities of stomach contents at dosing. This food–toxin interaction influences the availability of the toxin to the surfaces of the gut responsible for absorption [31] as well as affecting the rate of gastric emptying [32]. In fact, Carey and Merrill [33] stated that ad lib feeding is the “most poorly controlled variable in rodent bioassays”. Balancing the pros and cons of fasting, Kast and Nishikawa [32] concluded “for the doubtful advantage of eliminating influences on toxicity by factors related to the food, the test system has a distinctly increased absorption rate, with resulting unjustified higher toxicity values for many compounds”. To avoid the use of fasting, while eliminating the variability associated with ad lib feeding, we trialed a protocol whereby mice fasted overnight were fed a fixed amount of mouse food. Since the mice were hungry, they quickly ate the food offered, ensuring that each mouse had the same quantity of stomach contents at the time of dosing. The LD_50_ of mice fed 0.5 g of food prior to their STX.2HCl dose (2505 nmol/kg) was lower than those fed 1 g of food prior to their STX.2HCl dose (3618 nmol/kg). However, it is the LD_50_ using mice fed the smaller amount of food that is close to that determined using non-fasted mice (2850 nmol/kg) (Table 4). In contrast, the mice given 1 g (3618 nmol/kg) prior to their STX dose or unlimited access to food after fasting and their STX dose (3842 nmol/kg), had a higher LD_50_ than that normally observed. In these cases, due to fasting, the mouse was hungry, so it ate more than usual in one feeding event, making the stomach unusually full. For this reason, along with the fact that fasted mice eat 0.5 g of food quickly and reliably, it is the “fasted + 0.5 g food + toxin” feeding protocol that we recommend. It is interesting to note that the confidence limits of the LD_50_ using mice that were fasted prior to feeding a known amount of food are tighter than those with unlimited access to food (Table 4). This indicates less variability in the data which is consistent with other studies that have demonstrated variability due to ad lib feeding. 

The method used for oral administration is also a variable that has a big impact on toxicological studies. Gavage is often used but this is a difficult technique to master which can often result in rapid death due to aspiration into the lungs. Furthermore, a study by Craig and Elliott [34], using radiolabeled protein, showed that 38% of mice dosed by gavage did not receive their appropriate liquid dose, resulting in the authors concluding that “the common method of gavage feeding mice to assess absorption of orally ingested material can lead to artifacts not seen when the same agent is consumed under more natural circumstances” [34]. For shellfish toxins, it has long been recognized that gavage overestimates toxicity which has been thought to be due to the liquid gavage dose flowing around the semi-solid mass in the mouse stomach allowing rapid absorption by the upper duodenum [29]. This was consistent with our findings where the LD_50_ of STX.2HCl by gavage administration was 1.5 times lower than those determined by the other methods (Table 5). As an alternative to gavage administration, a method utilizing cream cheese has been commonly used. In this case, mice are trained to eat cream cheese over a few days after which time they are offered a small amount (150 mg) laced with the toxin dose. Under these circumstances, the entire dose is eaten by the mouse within 30 s without causing any stress to the animal. Although, under this protocol, the amount of cream cheese is very small it has been questioned whether it could affect the toxicity of shellfish toxins [35]. To investigate this possibility, the LD_50_ of STX.2HCl was determined using the cream cheese method (2455 nmol/kg) and was compared to that determined by feeding normal mouse food with the toxin dose directly applied to it (2505 nmol/kg). Results showed that the toxicity of STX.2HCl was unaffected by using cream cheese as the dosing matrix (Table 5).

## 4. Conclusions

By feeding mice diets containing STX.2HCl, a NOAEL could not be determined as a dose-dependent drop in food intake was observed. This was attributable to an effect on the palatability of the treatment diet.

The investigation of the effect of possible experimental variables on the toxicity of STX showed that the oral administration method and the quantity of the mouse stomach contents at the time of dosing were critical and must be standardized in the testing of PSP toxins. The best protocol for use in acute toxicity studies by oral administration is to fast the mouse before feeding 0.5 g of mouse food. The toxin dose can then be administered by adding it to the food offered or by giving it in a cream cheese matrix. 

In this study, the effect of dosing protocols on the toxicity of PSP toxins was investigated. Although further work is required to determine whether the effects observed for STX are seen for other classes of shellfish toxins, the consistent use of the suggested protocols by researchers involved in the toxicity testing of shellfish toxins would yield results that could be easily compared. However, as a minimum, it is important that the details of the methods used in the determination of toxicity are published alongside the data so that it is possible for other researchers to understand any source of ambiguity. Experimental details should include animal body weight, fasting time, dosing method and most importantly, feeding protocol.

## 5. Materials and Methods

### 5.1. Purity Assessment of Saxitoxin

STX was supplied by the Cawthron Institute (Nelson, New Zealand). This sample was calibrated against certified reference material from the National Research Council of Canada using an HPLC method adapted from Rourke, et al. [15,36]. The STX material was 99.8% STX, 0.16% decarbamoylSTX and 0.05% neoSTX. The STX stock solution had a concentration of 10.91 mg/mL STX.2HCl (29.3 µmol/mL), was dissolved in 3 mM HCl and stored at 4 °C. The quantities needed for each experiment were prepared gravimetrically using 3 mM HCl as the diluting solvent. 

### 5.2. Animals

Swiss albino mice, bred at AgResearch (Hamilton, New Zealand), were used for all experimental work. Female mice were used in all experiments, except for the one specifically exploring the effect of gender on STX toxicity. Mice were housed in a temperature-controlled room (21 ± 1 °C) with a 12 h light–dark cycle and with unrestricted access to water. All experiments were approved by the Ruakura Animal Ethics Committee established under the Animal Protection (code of ethical conduct) Regulations Act, 1987 (New Zealand; Project Numbers 15296 and 0563, approval dates 4 March 2021 and 31 March 2022, respectively).

### 5.3. Saxitoxin Feeding Study

#### 5.3.1. Preparation of Mouse Treatment Diets

Control diets, as well as those containing STX.2HCl, were based on Teklad Global 2016 mouse food pellets (Harlan UK, Bicester, UK) ground to a fine flour using a cyclone sample mill (Udy Corporation, Fort Collins, CO, USA). Portions of this material (50 g) were taken and the required amount of STX.2HCl was added to 40 mL of water. The water was added to the mouse food and the combination was mixed to form a paste which was used to form 50 small cookie-shaped portions of diet (approximately 1.5 cm diameter and 0.6 cm thickness). The mouse cookies were dried in a fan oven at 30 °C for 16 h. After drying, the 50 cookies of each batch were combined and weighed to allow the calculation of moisture content. The average water content was 10, 11, 12 and 12% for control, low STX, med STX and high STX treatment diets, respectively.

The STX.2HCl concentration of each treatment diet was determined by grinding one cookie in a mortar and pestle and then taking 200 mg aliquots for analysis by LC-MS/MS. The LC-MS/MS method used was based on that of Turner, et al. [37] which is described in full by Finch et al. [15]. Comparing the cookie STX.2HCl concentrations determined by LC-MS/MS with those that were expected based on the quantity of STX.2HCl added showed excellent correlation (Table 6) demonstrating diet homogeneity and stability of the STX.2HCl in diet.

Ambiguity can be incorporated into reports involving PSTs because concentrations can be expressed as either STX hydrate (free base) or STX dihydrochloride (STX.2HCl). Since it is often not specified which STX form the quoted concentration refers to, it is better to express concentrations on a molar basis [38].

#### 5.3.2. Experimental Protocol

A total of 16 female mice were individually caged and fed a control diet for two 1 h periods (9:00–10:00 a.m. and 3:00–4:00 p.m.) per day for three days. Food consumption and body weight were measured to ensure that each mouse had adapted to the mealtime feeding protocol. After three days, mice were randomly selected and assigned to treatment groups. Four treatment groups, each of four mice (individually caged) were fed between 9:00–10:00 a.m. and 3:00–4:00 p.m. for 7 days with the following diets. Group 1: control, Group 2: 3.67 µg/g STX.2HCl (9.86 nmol/g, STX low), Group 3: 4.57 µg/g STX.2HCl (12.3 nmol/g, STX med), Group 4: 6.65 µg/g STX.2HCl (17.9 nmol/g, STX high). All animals had unrestricted access to water throughout the experiment. Food consumption was measured by weighing food given at the start of each feeding period and weighing remnant food at the end of each feeding period. In addition, after each afternoon feed, the mouse body weights were measured, and each mouse was observed for any signs of toxicity. Using the daily food consumption and body weight data, the STX dose rate could be calculated for each mouse on each of the 7 days. After day 7, all mice were fed with control diets using the same feeding protocol. The health of mice and the measurement of food consumption and body weight continued for a further 7 days.

### 5.4. Acute Toxicity of Saxitoxin Using Different Experimental Protocols

Acute toxicity was determined according to the principles of OECD guideline 425 [39]. This guideline allows the minimum number of animals to be used while still yielding a robust determination of the LD_50_, including an estimate of confidence intervals. This method utilizes an up-and-down procedure whereby one animal is dosed with the concentration at one step below the estimated LD_50_. If this animal survives, the concentration dosed to the next animal is increased by a factor determined by the computer program associated with this method [40]. This factor is determined by estimating the slope of the dose–response curve. If the initial animal dies, the dose for the next animal is decreased by the same factor. Dosing is continued until four live-death reversals have been achieved. Mice were weighed immediately prior to dosing and test compounds dosed on a µg/kg basis. Aliquots of the test compounds were taken gravimetrically and diluted with 3 mM HCl prior to dosing. To avoid any diurnal variations in response, all dosing was conducted between 8:00–9:30 a.m. All mice were monitored intensively during the day of dosing. Mice that survived were monitored for a 14 day period which included regular measurement of body weight and food intake. After 14 days, the animals were euthanized using carbon dioxide inhalation and necropsied.

#### 5.4.1. The Effect of Mouse Bodyweight on the Toxicity of Saxitoxin by i.p.

Female mice weighing between 14 and 26 g were used in this experiment. An LD_50_ was determined for each 2 g weight band (i.e., 14 and 15 g mice in one weight band, 16 and 17 g mice in another weight band, etc.). I.p. administration of STX.2HCl was performed using a dose volume of 1 mL 3 mM HCl.

#### 5.4.2. The Effect of Dose Volume on the Toxicity of Saxitoxin by i.p.

Female mice of between 18 and 22 g body weight were used in this experiment. An LD_50_ was determined for STX.2HCl using dose volumes of 0.2, 0.5 and 1 mL 3 mM HCl.

#### 5.4.3. The Effect of Gender on the Toxicity of Saxitoxin by i.p. and Orally

Male or female mice of between 18 and 22 g body weight were used in this experiment. For i.p. administration a dose volume of 1 mL 3 mM HCl was used. Oral toxicity was performed by first pre-training mice to eat cream cheese. This was done by feeding cage groups of weanling mice small amounts of cream cheese twice daily. When mice were happy to eat this matrix (typically 2 days), they were individually caged and fed 200 mg of cream cheese on a watch glass. If mice ate this offering, they were ready to be dosed the following day and if not, the training process continued. On the day of dosing the STX.2HCl solution (<10 µL), was mixed with a small amount of cream cheese (150 mg) on a watch glass. The mice ate the laced cream cheese within 30 s.

#### 5.4.4. The Effect of Dosing Method on the Oral Toxicity of Saxitoxin

Female mice between 18 and 22 g body weight were used in this experiment. All mice were trained to eat cream cheese as described in Section 5.4.3. The ‘non-fasted’ mice had free access to food throughout the experimental period (before and after STX dosing). The mice that were ‘fasted + STX’ were fasted overnight and then given STX.2HCl laced cream cheese. Normal food was returned 4 h later. The mice that were ‘fasted + STX + unlimited food’ were fasted overnight, fed STX.2HCl in cream cheese and then immediately given unrestricted access to normal mouse food. The mice that were ‘fasted + 0.5 g food + STX’ were fasted overnight and then given 0.5 g of normal mouse food. When that had been consumed (<30 min), STX.2HCl in cream cheese was given. Normal food was returned 4 h later. The mice ‘fasted + 1.0 g food + STX’ followed the same protocol except they were given 1 g of food prior to STX.2HCl in cream cheese. 

## Figures and Tables

**Table 1:** The i.p. LD_50_ (nmol/kg and µg/kg) of STX.2HCl using mice of different bodyweights. Weight bands of 2 g were used.

BW (g)	14, 15 g	16, 17 g	18, 19 g	20, 21 g	22, 23 g	24, 25 g
LD_50_ nmol/kg	2903 (2673, 3007)	3196 (2878, 4176)	2372 (2051, 2857)	2673 (2312, 2857)	2372 (2051, 2857)	3007 (2513, 3441)
LD_50_ µg/kg	1080 (994, 1119)	1189 (1071, 1553)	882 (763, 1063)	994 (860, 1063)	882 (763, 1062)	1119 (935, 1280)

Numbers in brackets indicate 95% confidence limits.

**Table 2:** The i.p. LD_50_ (nmol/kg and µg/kg) of STX.2HCl in mice using different dosing volumes.

Dose Volume	LD_50_ (nmol/kg)	LD_50_ (µg/kg)
1.0 mL	27.8 (23.9, 96.4)	10.3 (0.89, 35.9)
0.5 mL	30.1 (27.8, 31.2)	11.2 (10.3, 11.6)
0.2 mL	31.2 (26.1, 35.9)	11.6 (0.97, 13.3)

Numbers in brackets indicate 95% confidence limits.

**Table 3:** The LD_50_ (nmol/kg and µg/kg) of STX.2HCl in male and female mice by i.p. and oral administration.

	i.p. Injection		Oral	
	Male	Female	Male	Female
LD_50_ (nmol/kg)	24.8 (21.6, 29.6)	24.0 (22.1, 24.8)	2900 (2673, 3007)	2850 (2475, 3410)
LD_50_ (µg/kg)	9.2 (8.0, 11.0)	8.9 (8.2, 9.2)	1079 (994, 1119)	1060 (921, 1269)

Numbers in brackets indicate 95% confidence limits.

**Table 4:** Oral LD_50_ (nmol/kg and µg/kg) of STX.2HCl in mice using different feeding regimes.

Feeding Regime	LD_50_ (nmol/kg)	LD_50_ (µg/kg)
Non-fasted	2850 (2475, 3410)	1060 (921, 1269)
Fasted + STX.2HCl	1504 (1312, 1777)	559 (488, 661)
Fasted + 0.5 g food + STX.2HCl	2505 (2339, 2506)	932 (870, 932)
Fasted + 1.0 g food + STX.2HCl	3618 (3341, 3675)	1346 (1243, 1367)
Fasted + STX.2HCl + unlimited food	3842 (3354, 5112)	1429 (1248, 1902)

Numbers in brackets indicate 95% confidence limits.

**Table 5:** The oral LD_50_ (nmol/kg and µg/kg) of STX.2HCl using different dosing methods.

Dosing Method	LD_50_ (nmol/kg)	LD_50_ (µg/kg)
Fasted + 0.5 g food + gavage	1671 (1479, 1945)	622 (580, 723)
Fasted + 0.5 g food + laced cream cheese	2505 (2339, 2506)	932 (870, 932)
Fasted + 0.5 g food + laced mouse food	2455 (2339, 2506)	913 (870, 932)

Numbers in brackets indicate 95% confidence limits.

**Table 6:** Measured and theoretical STX.2HCl (µg/g) concentrations in the treatment diets allowing the calculation of recovery.

Sample	Measured Concentration	Theoretical Concentration	Recovery (%)
Low	3.67	3.73	98
Med	4.57	4.97	92
High	6.65	6.21	107

## Data Availability

The data presented in this study are available on request from the corresponding author.
